# New Strains Intended for the Production of Inactivated Polio Vaccine at Low-Containment After Eradication

**DOI:** 10.1371/journal.ppat.1005316

**Published:** 2015-12-31

**Authors:** Sarah Knowlson, John Burlison, Elaine Giles, Helen Fox, Andrew J. Macadam, Philip D. Minor

**Affiliations:** Division of Virology, National Institute for Biological Standards and Control, Potters Bar, Hertfordshire, United Kingdom; Columbia University, UNITED STATES

## Abstract

Poliomyelitis has nearly been eradicated through the efforts of the World Health Organization’s Global Eradication Initiative raising questions on containment of the virus after it has been eliminated in the wild. Most manufacture of inactivated polio vaccines currently requires the growth of large amounts of highly virulent poliovirus, and release from a production facility after eradication could be disastrous; WHO have therefore recommended the use of the attenuated Sabin strains for production as a safer option although it is recognised that they can revert to a transmissible paralytic form. We have exploited the understanding of the molecular virology of the Sabin vaccine strains to design viruses that are extremely genetically stable and hyperattenuated. The viruses are based on the type 3 Sabin vaccine strain and have been genetically modified in domain V of the 5’ non-coding region by changing base pairs to produce a cassette into which capsid regions of other serotypes have been introduced. The viruses give satisfactory yields of antigenically and immunogenically correct viruses in culture, are without measurable neurovirulence and fail to infect non-human primates under conditions where the Sabin strains will do so.

## Author Summary

New polio vaccines will be needed to safeguard global eradication: Sabin strains are known to evolve to fill the niche left by wild-strains so their long-term use is incompatible with eradication; most current inactivated vaccine is made from wild polioviruses so that production presents a significant biosecurity risk. We have developed new strains for Inactivated Polio Vaccine (IPV) production with negligible risk to the human population should they escape. Sabin’s live-attenuated vaccines are variants of wild strains selected by the use of unnatural cell substrates, hosts and growth conditions. Unsurprisingly these variants evolve back towards wild-type properties during replication in, and transmission between, their natural hosts. An understanding of the molecular basis of these pathways led us to design novel vaccine strains that are very highly attenuated and arguably cannot replicate in people and whose opportunities for reversion during replication in cell culture are severely restricted. At the same time, the strains can feasibly be produced on a large-scale and they are as immunogenic as current IPV. These attributes allow for safe vaccine production in the post-eradication world.

## Introduction

The Global Polio Eradication Initiative (GPEI) is the biggest and most ambitious health programme aimed at a single disease in history and is very close to eliminating naturally occurring wild poliovirus from the planet [1]. It has involved the extensive use of both the live attenuated vaccines that can revert to a wild type phenotype [2], and inactivated polio vaccines (IPV) whose production in the main currently requires the growth of very large amounts of virulent wild type poliovirus [3]. The vaccines are therefore a possible source for the re-emergence of poliomyelitis when poliovirus has been eradicated.The manufacture of inactivated polio vaccine by new global producers is being encouraged to improve supply and reduce cost [4,5] and this increases the possibility of escape or release of large amounts of virus as a result of accident or because the facilities become terrorist targets. WHO has therefore encouraged novel approaches to vaccine production including the use of the attenuated but genetically unstable Sabin vaccine strains for production of IPV on the grounds that this will be safer than use of the wild type strains [3,4,5,6]. Here we describe a more radical approach to safer IPV production exploiting understanding of the attenuation of poliovirus for humans and animals to design and characterise strains that are genetically stable and able to grow in cells in culture but cannot revert to a form that can paralyse people or, arguably, grow in the human gut. Should such a strain escape the production plant it will therefore be harmless.

**Fig 1:**
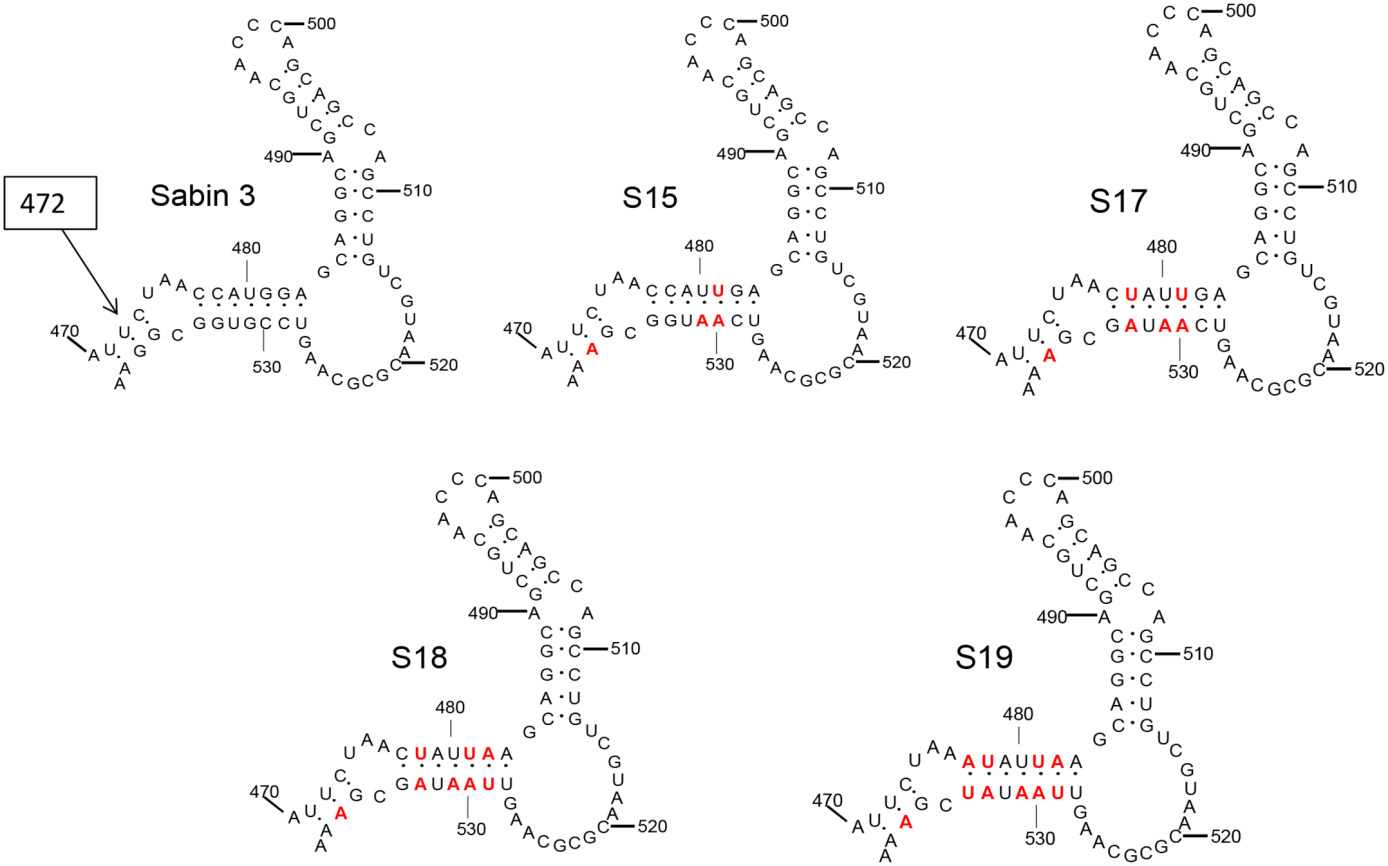
Base paired stem loop structures in domain V of the 5’ noncoding region of type 3 poliovirus. Arrow indicates base 472, which is a U in the Sabin type 3 strain but a C in revertants. Red base pairs show substitutions made; either of GC to AU, or mismatches or GU to AU pairs.

Polioviruses occur in three serotypes, 1, 2 and 3 and are positive stranded RNA viruses of the picornavirus family with a genome of approximately 7500 nucleotides. A single large open reading frame is preceded by a highly structured 5’ non coding region of about 750 bases containing an Internal Ribosomal Entry Site (IRES) through which the virus initiates protein synthesis [2]. All three of the attenuated vaccine strains developed by Sabin and used in the eradication programme contain mutations that weaken the three or seven base pair adjacent stems between bases 470 to 483 and bases 528 to 538 of a particular region of the IRES designated domain V [2]. This is shown in Fig 1 for the type 3 strain where the base pair between nucleotides 472 and 537 is a UG in the Sabin strain rather than a CG as found in wild type strains. The U rapidly reverts to a C in vaccinees increasing the thermodynamic stability of the structure, and there is a concomitant increase in neurovirulence of the virus relative to the vaccine [2]. The type 1 and type 2 strains also revert mutations or insert compensating changes in the same domain and by day 28 all vaccinees who are excreting type 2 and 3 viruses and over 80% of those excreting type 1 virus are excreting revertants, indicating a strong selection pressure in the gut against mutations in domain V [2]. We believe that a virus in which this region could not revert would be unable to grow in the human gut. In any event it would be stably attenuated. We have previously shown that the temperature sensitivity of growth in certain cell lines is a correlate of attenuation caused by mutation of this region of domain V [7].

The thermodynamic stability of domain V can be adjusted by introducing single base changes or by manipulating base pairs, for instance replacing triple hydrogen bonded GC base pairs with weaker double hydrogen bonded AU pairs. We have previously reported that a structure modified by base pair exchange so that it has the same thermodynamic stability as the Sabin type 3 vaccine strain (S15 in Fig 1) has a similar level of attenuation as the Sabin type 3 vaccine strain [7]. As the base pair requires two simultaneous mutations to revert to the more stable wild type GC (any other intermediate pairing being less stable), the virus is genetically more stable on passage. For maximum genetic stability, however, any single hydrogen bonded GU base pairs must be replaced with Watson-Crick pairs so that they cannot increase the thermodynamic stability by a single mutation. By adjusting relevant base pairs a structure that is predicted to be both minimally stable thermodynamically and maximally stable genetically can be produced. We have constructed a series of such recombinant genomes and investigated the properties of viruses recovered from them including their neurovirulence, temperature sensitivity and genetic stability on passage. Selected strains were further studied for virus yield, infectivity in a primate model and immunogenicity in a rat model compared to strains currently used in production of IPV.

## Results

### Viruses with modified domain V structures

The type 3 vaccine strain was used as a starting point as there is a great deal of data on its attenuation and reversion. Fig 1 shows the successively weaker structures that have been generated, designated S15, S17, S18 and S19.

### Growth and properties of type 3 polioviruses with modified domain V structures

**Fig 2:**
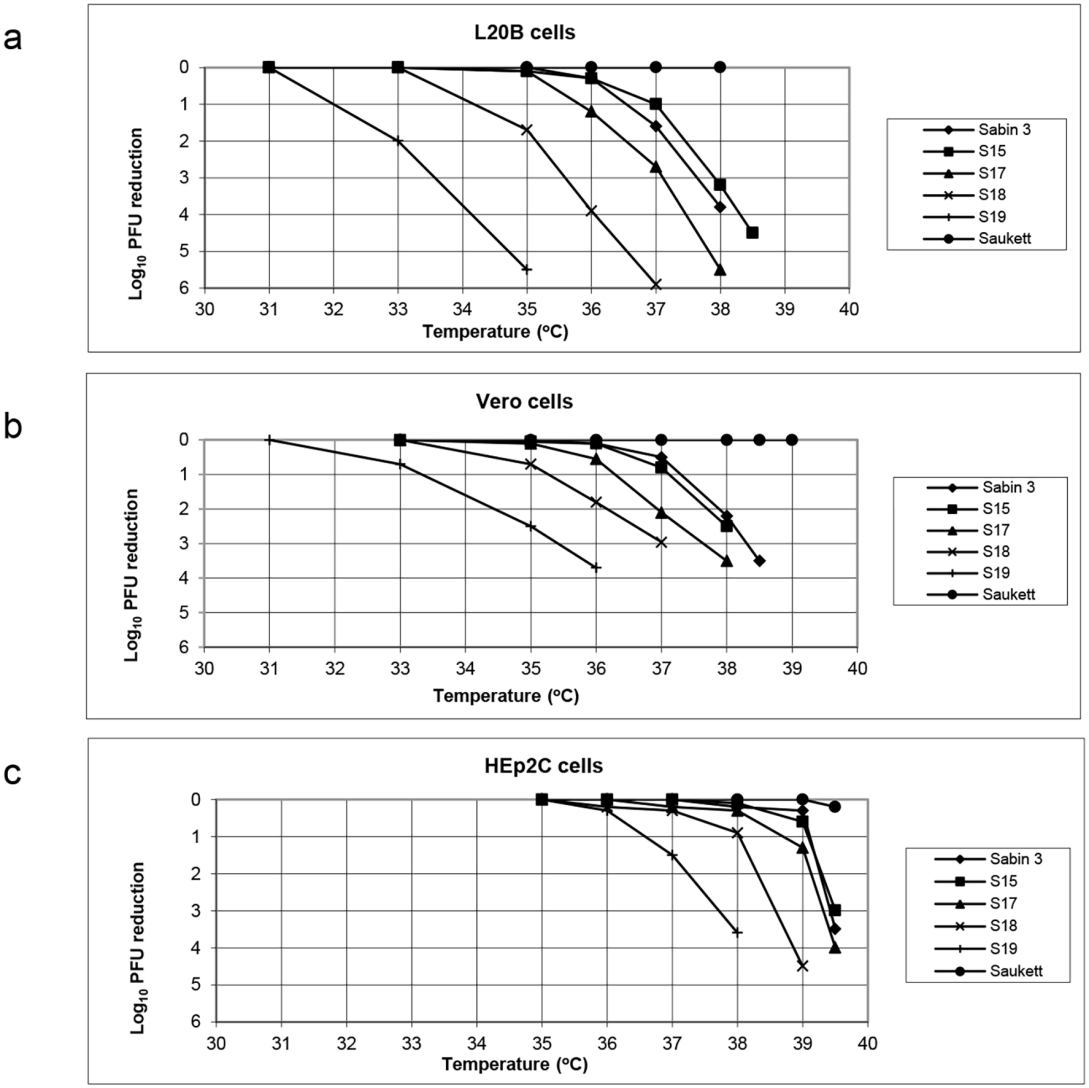
Reduction in plaques formed by modified viruses at different temperatures compared to permissive temperature. a, L20B cells, b, Vero cells, c, Hep2c cells. The type 3 strains Sabin, Saukett, S15, S17, S18 and S19 were examined.

Virus strains based on the type 3 Sabin vaccine strain containing the modified domain V sequences corresponding to S15, S17, S18 and S19 were recovered by transfection. Their growth at different temperatures and in different cells was assessed as shown in Fig 2 for L20B cells (a mouse L cell line stably transfected with the human receptor for poliovirus [8]), Vero cells and Hep2C cells. The results are expressed as the log_10_ reduction in plaque titre at various temperatures relative to that found at the permissive temperature (31°C for L20B and Vero or 35°C for Hep2C). The unmodified type 3 Sabin vaccine strain and the wild type 3 Saukett strain used in the current production of inactivated polio vaccine (IPV) were included for comparison. As previously reported S15 showed similar characteristics to the Sabin type 3 vaccine strain [7]. In L20B cells both viruses grew well at 35° but showed a 1–2 log_10_ reduction in titre at 37° and a 3–4 log_10_ reduction at 38°. In Vero cells both viruses showed a 0.5–0.8 log_10_ reduction at 37° compared to 33° or 35° and a 2.0 log_10_ reduction at 38°. In Hep2c cells growth was not affected below temperatures of 39.5°. The growth of the other viruses was increasingly temperature sensitive in all cells as the domain V structure was progressively weakened, with S19 showing a 2 and 0.8 log_10_ reduction between 31 and 33°C in L20B cells and Vero cells respectively and a reduction of 1.5 log_10_ between 35 and 37°C in Hep 2c cells.

The neurovirulence of the strains was assessed by intraspinal inoculation of transgenic mice carrying the human receptor for poliovirus as used for the regulatory testing of oral polio vaccine [9],using a dilution series to determine the 50% paralytic dose. The results are shown in Table 1. The Sabin type 3 strain and S15 gave PD50s of 3.6 and 3.7 log_10_ CCID_50_ (Cell Culture Infectious Dose 50%), S17 gave a PD50 of 7.4 log10, about 4 log10 more than S15, and it was impossible to inoculate sufficient virus for any other strain to paralyse 50% of the mice. Neither S18 nor S19 paralysed any animal and the data demonstrate a sharply increasing degree of virus attenuation as the domain V structure is progressively weakened culminating in S19.

**Table 1:** Attenuation phenotypes in transgenic mice of viruses modified in domain V of Sabin type 3 poliovirus.

Virus	PD_50_ in log_10_ CCID_50_ (number paralysed/total)
Sabin 3	3.6
S15	3.7
S17	7.4
S18	>8.4 (0/16)
S19	>8.2 (0/16)

### Seed viruses for production of Inactivated Polio Vaccine (IPV) derived from S19

When S18 was passaged ten times in Vero cells at 33, 35 or 37°C no mutations were induced in domain V but a range of mutations in protein 2A occurred as expected from previous published work[10]Protein 2A is involved in the shut off of cap dependent protein synthesis in poliovirus infected cells. We have previously shown that in certain cells, including Vero, the effect of mutations in domain V of the 5’non coding region can be suppressed by a surprisingly extensive series of coding mutations in protein 2A; the basis for this phenomenon remains unclear. The mutations do not affect neurovirulence in any model or growth in cells of human origin [10]. Substitution of an asparagine by a serine at amino acid 18 in protein 2A (N18S) occurred independently several times and the mutation occurs at a base close to a convenient restriction site. While other equally satisfactory suppressors could have been chosen, viruses based on S19 were therefore constructed that included the N18S substitution to allow good growth in Vero cells.

**Fig 3:**
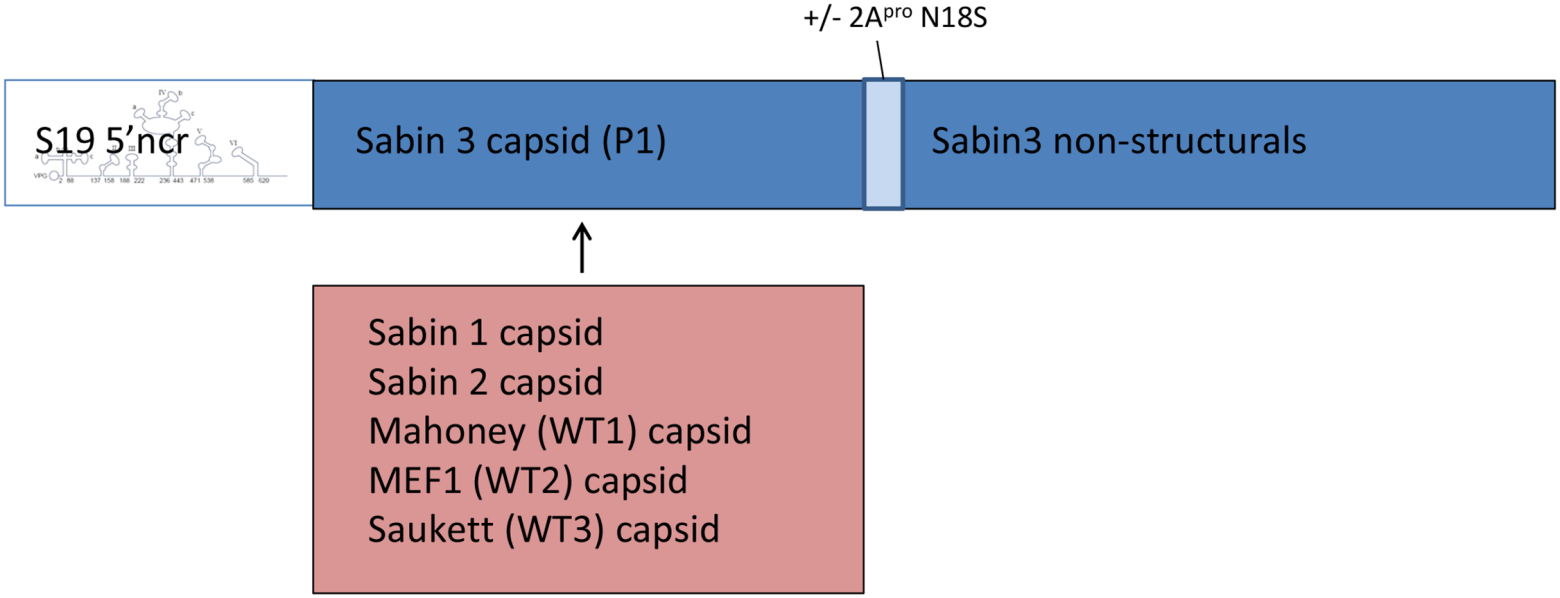
Structure of S19 viruses. The genetic cassette consisted of S19 where the capsid region was substituted by that for Mahoney or Sabin 1, MEF1 or Sabin 2 or Saukett. A parallel series of viruses was made including a mutation in the 2A protein where an asparagine at residue 18 was replaced by a serine to allow better growth in Vero cells.

S19 and S19/N18S were used as the basis for construction of chimeric viruses in which the capsid proteins were replaced with those of other wild type or Sabin vaccine strain polio viruses. The wild type strains used were the type 1 Mahoney strain, the type 2 polio virus MEF1 strain and the type 3 Saukett strain all of which are used in current classical IPV production. The same genetic backbone was used because there is a good understanding of the properties of the type 3 strain and its attenuation, and the donors of the capsid regions were chosen because use of the Sabin vaccine strains for IPV production is being encouraged with a product licensed in Japan for two years, whilst the clinical properties of vaccines produced from the wild type strains used in current vaccine programmes are well known. The capsids are the immunogenic component of IPV. The virus constructs are summarised in Fig 3.

### Neurovirulence of the S19 based vaccine seeds

The neurovirulence of the strains was assessed by intraspinal inoculation of transgenic mice carrying the human receptor for poliovirus. The results are shown in Table 2. The parental Sabin Vaccine strains had 50% paralytic doses of 2.25, 5.9 and 3.6 log_10_ 50% Cell Culture Infectious Dose (CCID50) for the type 1,2 and 3 strains respectively. These figures are consistent with those expected from commercial vaccines [9]. The PD50s for the wild type strains were much lower as expected being 0.5, 0.7 and 0.8 for the Mahoney, MEF1 and Saukette strains respectively.

**Table 2:** Attenuation phenotype in transgenic mice of wild type and S19 derivative strains.

Virus	PD_50_ log_10_ CCID_50_
Mahoney	0.5
Sabin 1	2.25
MEF1	0.7
Sabin2	5.9
Saukett	0.8
Sabin3	3.6
S19/S1P1	>8.15 (0/8)
S19/MahP1	>8.10 (0/8)
S19/S2P1	>8.15 (0/8)
S19/MEF1P1	>8.2 (0/16)
S19	>8.2
S19/SktP1	>8.45 (0/8)
S19/S1P1/N18S	>7.8 (0/8)
S19/MahP1/N18S	>7.95 (0/8)
S19/S2P1/N18S	>8.85 (0/8)
S19/MEF1P1/N18S	>8.05 (0/8)
S19/(S3P1)/N18S	>8.1 (0/8)
S19/SktP1/N18S	>7.7 (0/8)

None of the constructs based on S19 produced any paralysis in any mouse at the highest dose that could be injected, which ranged from 8.10 to 8.45 log_10_ CCID50. Given the 3-4log_10_ difference between PD50s of S15 and S17 (Table 1) these results suggest the true PD50s of the S19 strains are in excess of 12.0 log_10_ CCID50.

In contrast to the seeds based on unmodified S19, those containing the N18S mutation in 2A gave high yields in Vero cells without the need for further adaptation.Previous studies have shown that the mutations do not affect neurovirulence in the tests applied [10] and as can be seen from Table 1 no S19 N18S derived strain caused paralysis at the highest dose that could be inoculated.

**Fig 4:**
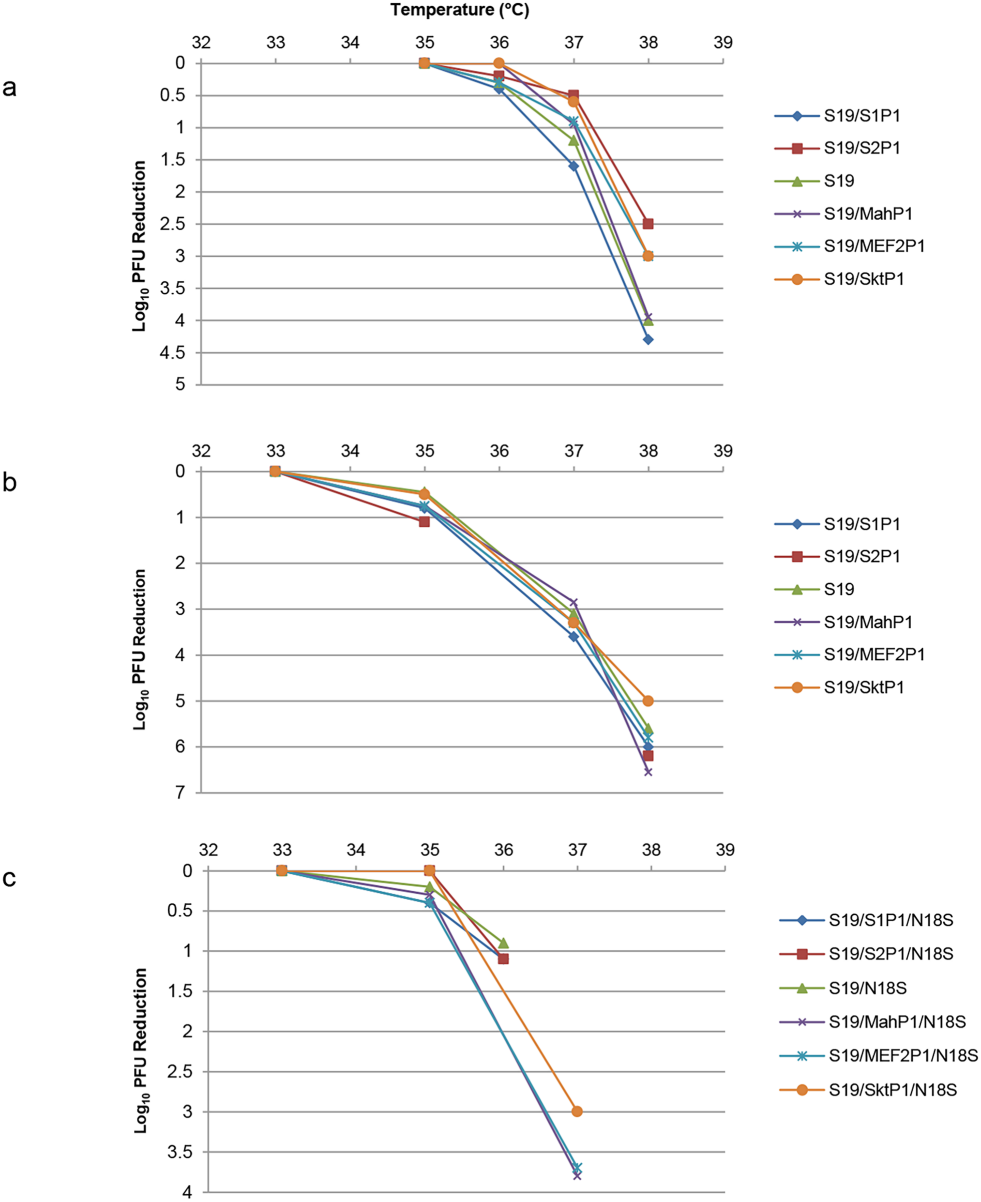
Reduction in plaques formed by S19 viruses shown in Fig 3 compared to permissive temperature: a, Hep2c cells, b.MRC5 cells. c shows the reduction in plaques formed by the S19 N18S series of viruses in Vero cells.

The novel capsid could have affected the growth properties of the viruses in cells. Growth was studied at a range of temperatures in Vero, Hep2c and MRC 5 cells and the data in Hep2c and MRC5 cells are given in Fig 4a and 4b respectively. For all of the S19 derived strains the curves are super-imposable so that the capsid had no effect on the temperature sensitive growth characteristics. Some of the Sabin strains are known to have mutations conferring a temperature sensitive phenotype in the capsid proteins; however the low temperatures at which the S19 structure has its effect are all fully permissive for the Sabin capsid region mutations, so a lack of effect is to be expected. The same lack of effect of the capsid regions was found for S19 N18S derived strains in Vero cells as shown in Fig 4c. The S19 derived strains without the N18S mutation grew poorly in Vero cells.

### Stability of the S19 based vaccine seeds on passage in cell culture

The S19 N18S seeds were passaged 10 times in Vero cells and in MRC5 cells at 33°C. No mutations were detected in domain V by Sanger sequencing and the virulence of the strains as measured by PD50 assay in transgenic mice was unchanged with no mice paralysed at the highest dose that could be inoculated (Table 3). Mutations were occasionally found as mixtures in the capsid region and in one instance in the 5’NCR at base 128 in S19 MEF1P1 N18S passaged in Vero (S1 Table). We have previously shown that the Sabin type 3 strain reverts completely both genetically and phenotypically when passaged at 37° [7].The mutations that had been deliberately inserted in the S19 series did not change on passage.

**Table 3:** Attenuation phenotype of passaged S19/N18S strains in transgenic mice.

Strain	Pass 10 MRC5 cells PD_50_ log_10_	Pass 10 Vero cells PD50 log10
S19/MahP1/N18S	>7.95 (0/8)	>8.1 (0/8)
S19/ MEF1P1/N18S	>8.05 (0/8)	>8.15 (0/8)
S19/ SktP1/N18S	>7.75(0/8)	>7.65 (0/8)
S19/ S1P1/N18S	>7.8 (0/8)	>7.95 (0/8)
S19/ S2P1/N18S	>8.85 (0/8)	>7.85 (0/8)
S19/ S3P1/N18S	>8.1 (0/8)	>7.7 (0/8)

### Yield of virus

Given the temperature sensitivity of the S19 strains the ease with which the viruses can be grown is crucial to their suitability as seed strains. One step growth yields at 33°C in MRC-5 or Vero cells for the relevant seeds are given in Table 4 in comparison to the wild type strains currently used in conventional production. The S19 strains took longer than the wild type strains, but in all cases complete cpe was reached in 24–48 hours. The effect of passage on yield was not investigated for these constructs. The Sabin strains are not yet widely used in production for IPV but are expected to give slightly lower yields. The yields in MRC-5 cells are slightly lower by 0.2 and 0.8 log_10_ for the S19/MahoneyP1 and S19/MEF1P1 strains respectively while the yield for the Saukett strain was slightly higher by 0.15 log_10_. The strains intended for Vero based production gave titres about 0.5 log_10_ lower in Vero cells than the corresponding wild type. These differences are small but probably real and it is likely that they could be eliminated by optimising the conditions of growth in the course of process development by a manufacturer. The growth of the strains is compatible with production.

**Table 4:** Single step growth yields at 33° in Vero and MRC-5 cells.

Virus	MRC-5 log_10_ CCID_50_/ml	Vero log_10_CCID_50_ /ml
S19/MahP1	9.0	
S19/S1P1	8.45	
S19/MEF1P1	8.6	
S19/S2P1	8.5	
S19/SktP1	8.85	
S19/S3P1	8.65	
S19/MahP1/N18S		8.8
S19/S1P1/N18S		8.9
S19/MEF1P1/N18S		8.8
S19/S2P1/N18S		8.15
S19/SktP1/N18S		8.45
S19/S3P1/N18S		8.95
Mahoney	9.2	9.35
MEF1	9.4	9.3
Saukett	8.7	9.05

### Immunogenicity of S18 derived strains

Mature infectious poliovirus expresses a different antigen (D antigen) to the empty capsid (C antigen) and the potency of IPV is usually expressed in D antigen units measured by ELISA relative to an International reference established by WHO. The potency of IPV is also assessed by immunising rats and comparing the responses to those produced by the same reference. Different strains of poliovirus differ in the immune response generated for the same mass expressed in D antigen units. Assays were carried out using the S18 versions of the strains which have identical capsid proteins to the analogous S19 strains. Virus preparations were inactivated with formalin imitating the process used commercially [11]. The D antigen content of preparations of inactivated S18 derived strains were measured by ELISA by methods used in house to assay commercial IPV batches [12]. Rats were then immunised with the S18 strains and the wild type IPV International Standard over a similar range of D antigen content [13]. The amount of S18 strain D antigen required to generate an immune response equal to the reference was expressed as a ratio and is shown in Table 5. The S18 strains gave a ratio of very close to 1 and were therefore of equivalent immunogenicity per unit antigen to the unmodified strains, except for the type 2 Sabin capsid strain which is known to be less immunogenic and gave a ratio of about 0.1 [14]. The S18 strains therefore gave figures consistent with the capsid regions they expressed.

**Table 5:** Immunogenicity after inactivation relative to reference strains.

Virus	Relative potency (95% CI)
S18/S1P1	1.2 (0.6–2.3)
S18/MahP1	1.5 (0.8–3.0)
S18/S2P1	0.1 (0.04–0.2)
S18/MEF1P1	1.0 (0.4–2.4)
S18/S3P1	1.6 (0.8–3.2)
S18/SktP1	1.6 (0.8–3.3)

### Infectivity of S19 strains in primates

Humans are more readily infected by the oral route than primates [15]. However an animal model was established in which cynomolgus macacques were fed polio virus and monitored for up to 49 days for virus excretion and then for seroconversion. The results are shown in Table 6 for the Sabin type 3 strain, S19 and the wild type S19 derivatives. Only the Sabin 3 strain showed signs of infecting the animals, being excreted for a period of 14 to 42days by all four animals given 10^11^pfu in experiment 1 and by two of four animals given 10^9.5^pfu in the second experiment. The dose in vaccines for human use is in the region of 10^5^ depending on the serotype Where animals were not infected virus was detected in the stool at declining titers for less than six days consistent with the dose given; where infection was successful the titres were of the order of 100 pfu per gram of stool, which is about two orders of magnitude less than that found in humans.

**Table 6:** Infectivity after oral administration of virus to primates.

Virus	Replication	Seroconversion
Sabin 3 (10^11^ pfu)	4/4	2/4
Sabin 3 (10^9.5^ pfu)	2/4	1/4
S19 (10^11^ pfu)	0/4	0/4
S19/MahP1 (10^11^ pfu)	0/4	0/4
S19/MEF1P1 (10^11^ pfu)	0/4	0/4
S19/SktP1 (10^11^ pfu)	0/4	0/4

Only animals infected with the Sabin 3 strain seroconverted. The data were consistent with a failure to infect with any of the S19 derived strains and provide some support to the view that the strains would not infect the human gut.

However the virus excreted after infection with the Sabin 3 strain retained the U at base 472 throughout the excretion period, whereas it would have rapidly mutated to a C in humans. Other mutations were introduced into the virus excreted by the animals given the Sabin type 3 strain including mutations in protein 2A towards the end of the monitoring period. Such mutations have not been reported in human studies [2].

## Discussion

We have constructed viruses which are extremely attenuated and genetically stable in cell culture by rational design based on understanding of the attenuation of the Sabin vaccine strains of poliovirus. The viruses grow to titres acceptable for IPV production under appropriate conditions of temperature and cell substrate and have the same antigenic properties based on reactions with panels of monoclonal antibodies in ELISA as well as in regulatory assays for antigen and immunogen content as the strains from which their capsids were derived.

It is intended that the S19 strains should be used for production as they represent the most attenuated case; however the properties of S18 make it also suitable for safe production. Should the S19 strains revert by insertion of a double mutation the resulting virus would be S18 like and therefore also safe, providing a margin of security; in fact it would require six paired mutations to produce a virus that was as virulent as the current Sabin strains. It can be argued that the attenuating mutations could be removed by a single recombination event involving a poliovirus or a type C enterovirus. Recombination with a poliovirus that could compensate for the defective 5’non coding region is unlikely since all the production strains have the same basic genetic structure and could not complement each other and the use of other poliovirus strains will be highly constrained after eradication and containment. There is no reason why the viruses should be exposed to an enterovirus species C in the laboratory if good practice is followed, and the most likely exposure would be if a worker was already infected with such a virus and then became exposed to an S19 strain described here. The strains are very highly and stably attenuated and will not cause paralysis. However we believe that there is strong indirect evidence that the strains will be unable to grow in the human gut. This is based in part on their very low fitness in vitro and in vivo, but more importantly on the well documented reversion of the 5’ non coding mutations in vaccinees where recipients of vaccine revert the mutations within a very short time of immunisation, implying a very strong selection pressure against the disruption of the structure (see reference 2 and references therein). The strains are not able to accomplish reversion of this type, requiring multiple simultaneous mutations to regain fitness. The data on oral infectivity in an animal model are consistent with the view that the viruses are of low infectivity by the oral route, although the model is not a perfect reflection of human infection. This suggests that recombination in the human gut with a virus that has an established infection would be difficult and unlikely irrespective of the comparative fitness of a product of such an event. The inferred inability to grow in the human gut makes the strains safe for production; should someone be accidentally exposed through a release from a production facility they will not become infected and will not pass the strain onto others. We consider that the viruses are intrinsically safer and should be fit for use at lower containment than the wild type or Sabin vaccine strains.

The recently issued WHO global action plan to minimise poliovirus facility associated risk after type specific eradication of wild polioviruses and sequential cessation of OPV use (GAP III)specifies poliovirus containment and risk mitigation strategies as polio is eradicated[5]. Vaccine strains, defined as any strain licensed for use as a live vaccine, i.e. the Sabin strains, are contained by different criteria to wild type strains. Wild type strains encompass everything else including any virus with a wild type capsid or vaccines not licensed for human use such as Cox, CHAT and other strains [5]. The strains described here would be classified as wild type by these criteria as they are not and cannot be licensed as live vaccines on grounds of efficacy at least. However GAP III also leaves open the option of assessing new derivatives by an expert panel that will compare the novel strains to the Sabin strains with respect to degree and stability of attenuation, potential for person to person transmission and neurovirulence in animal models to define the containment required which is not specified. GAP III is also described as an evolving document.

Vero cells are the preferred cells for production of IPV as they are robust and easier to adapt to large scale than alternatives such as the human diploid MRC5 cells. However mutations are selected rapidly when the S19 strains are grown in Vero cells; consequently seed constructs were made with one suitable mutation that allowed good growth. The mutation did not affect virulence or growth in other cells. Moreover mutations in 2A have not been seen in viruses excreted by recipients of vaccine [2]. For these reasons we believe that they do not affect the phenotype of S19 so far as human subjects are concerned or affect the considerations of containment required in the use of S19 derived seed in IPV production.

Finally, historically live attenuated viral vaccines have been extremely successful but their development has been based on serendipitous identification linked to careful clinical evaluation of their suitability rather than rational manipulation based on understanding of the virus [16]. In contrast the strains described here were derived by application of the molecular biological understanding of the physiology and attenuation of the polio strains used in live vaccines. The properties were correctly predicted a priori.

## Materials and Methods

### Cells and viruses

Hep 2c, Vero, MRC-5 and L20B cells were grown as previously described [17]. Hep2c cells were from stocks maintained at NIBSC since the 1970s, Vero cells originated from ATCC; MRC-5 cells were originally derived at NIBSC in 1968 and L20B cells were originally supplied by Dr Vincent Racaniello, Columbia University New York; the cells used here were part of a cell bank maintained at NIBSC to supply WHO laboratories of the Polio Laboratory Network. Viruses were grown on confluent monolayers at the relevant temperature at a multiplicity of infection of 10. Cell sheets were incubated in serum free medium supplemented with antibiotics until complete cytopathic effect was seen, when the flasks were frozen and thawed three times followed by removal of cell debris by centrifugation. To establish single cycle growth curves replica MRC-5 or Vero cell monolayers in 6-well plates were inoculated with viruses at multiplicities of infections (MOI) of 10. Infected cell sheets were incubated in MEM containing antibiotics but no serum at 33°C for different periods then frozen at -70°and thawed, three times, and cell debris removed by centrifugation. Supernatants were titrated in HEp2C cells at 33°C by CCID50 assays in 96-well plates.

### Temperature sensitivity

Viruses were assayed by plaque-formation at different temperatures in Hep2C cells, Vero cells and L20B cells. Temperatures were controlled by incubation of inoculated plates in sealed plastic boxes submerged in water baths whose temperatures fluctuated by <0.01°C. All viruses were assayed at least twice and control viruses with known phenotypes were always included for validation. The range of temperatures over which variation in domain V stability influences viral growth depends on the cell substrate used [17,18] being the lowest in L20B cells and highest in HEp2c cells.

### Virus passage

MRC-5 or Vero cell monolayers in 25cm2 flasks were inoculated with viruses at multiplicities of infections (MOI) of 1. Infected cell sheets were incubated in MEM containing antibiotics but no serum at 33°C or 37°C until complete cytopathic effect (cpe) was apparent. Flasks were frozen at -70°and thawed, three times, and cell debris removed by centrifugation. Supernatants were used for further passage, as above, or for biological and molecular analysis.

### Construction and recovery of variants

Derivation of the Sabin 3 cDNA clone and construction of S15 have been described previously [7,19]. S17, S18 and S19 were constructed by PCR mutagenesis; mutations introduced into each sequence are shown in Fig 1. For each plasmid, three fragments of the 5’ noncoding region of Sabin 3 were amplified by PCR using primers incorporating the necessary sequence changes, located at nucleotides (a) 31–50 and 471–489, (b) 471–489 and 522–540 and (c) 522–540 and 755–778. The three overlapping fragments (a)-(c) were gel-purified, mixed and re-amplified with outer primers then the 747bp fragment comprising the mutated 5’ noncoding region was cloned into pCR2.1 (Invitrogen) and sequenced. M1uI-SacI (279–751) fragments with correct sequences were ligated into Sabin 3 clones lacking the SacI-SacI (751–1900) fragment. Full-length infectious clones were generated by addition of a partial SacI/SmaI (2768) fragment.

In order to replace the P1 coding region a SacII site was introduced without coding change into the S19 clone at nucleotides 3408–13. Capsid regions from Sabin 1, Sabin 2, Mahoney, MEF-1 and Saukett were then introduced precisely using standard PCR methods. Residue 2A-18N was mutated to S using standard methods. All clones were verified by sequencing.

Viruses were recovered by transfection of HEp2C monolayers with >2μg T7 transcripts [20] followed by incubation at 33°C for 24–48 hours, by which time complete cytopathic effect was apparent. Sequences of all mutants were confirmed following RNA extraction and RT-PCR.

### Intraspinal inoculation of TgPVR mice

The TgPVR mouse experiments were performed under Home Office licences PPL 80/2478 and PPL 80/2050 which were reviewed and approved by the NIBSC animal ethical committee before submission. High titre virus stocks were prepared by inoculating HEp2C monolayers in 75cm2 flasks (four per virus) at multiplicities of infections (MOI) of 10 and incubating at 33°C until complete cytopathic effect (cpe) was apparent. Flasks were frozen at -70°and thawed, three times, and cell debris removed by centrifugation. Supernatants were centrifuged through 10ml cushions of 30% sucrose in PBS ‘A’ in an SW28 rotor at 25,000 rpm overnight. Pellets were resuspended in a total of 1 ml PBS ‘A’ and passed through a 0.45μ sterile filter prior to inoculation.

Intraspinal inoculation was performed essentially according to the standard operating procedure “WHO neurovirulence test of type 3 live poliomyelitis vaccines (oral) in transgenic mice susceptible to poliovirus” available from the WHO (Coordinator, Quality Assurance & Safety: Biologicals, World Health Organisation, 1211 Geneva 27, Switzerland) except that higher doses and fewer mice per dose were used. Briefly, 6–8 week old TgPVR mice in groups of 8 (weight and sex-matched) were sedated and inoculated into the lumbar region of the spinal cord with 5μl of each dose and observed for occurrence of paralysis for up to 14 days. Mice with paresis/paralysis were scored positive and mice surviving for 14 days with no clinical signs were scored negative.

### Oral infectivity

The experiments were performed under Home Office licence PPL 80/2060 and were specifically approved by the NIBSC animal ethics committee.Cynomolgus macaques (between 1.5 and 3.5 kg) were trained to accept small volumes of fruit cordial by mouth from a syringe on an appropriate signal. The monkeys were then caged in groups of four and starved for at least 12 hours prior to oral inoculation. On day 0 monkeys were given 2ml virus inoculum mixed with fruit cordial, by mouth from a syringe. Separate faecal samples (5-10g) for virus isolation were collected from each animal on days: -1, 1, 2, 3, 4, 5, 6, 7, 10, 14, 17, 21, 28, 35, 42 & 49. For sample collection, animals were caged individually at an appropriate time of day, with a clean lined collecting tray placed under the cage, until samples are produced. Blood samples (5-10ml) were taken on days: -1, 7, 14, 21, 28, 42.

Faecal samples were processed for virus isolation by adding 1g to10 ml PBS containing 1g of glass beads and 1 ml chloroform in a 50mL Falcon tube. Tubes were shaken vigorously at 4°C for 20 minutes using a mechanical shaker then centrifuged for 20 minutes at 1500 x g in a refrigerated centrifuge. Supernatants were stored at -20°C prior to virus titration and isolation.

Faecal virus titres were measured by plaque assay in Hep2C cells. Virus stocks, prepared by inoculating HEp2c cells, were characterised by (Sanger) nucleotide sequencing. Neutralising antibodies in monkey sera were determined using standard protocols.

### Immunogenicity

Immunogenicity was assessed using Pharmacopieal methods established at NIBSC for the release of IPV lots. Viruses were inactivated by formaldehyde treatment (0.01% at 37°C, for twelve days [11]), D antigen content was measured by ELISA [12] and immunogenicity was assessed in Wistar rats [13].The neutralising antibody responses to a range of antigen doses were statistically compared to those elicited by a concurrently tested International Standard preparation to derive a relative potency: a potency of 1.0 indicates equivalence; vaccine lots are acceptable for release if the statistical 95% confidence interval of the assay of their potency includes 1.0.

### Ethics statement

All animal experiments were performed under licenses granted by the UK Home Office under the Animal (Scientific Procedures) Act 1986 revise 2013 and reviewed by the internal NIBSC Animal Etics committee. The TgPVR mouse experiments were performed under Home Office licences PPL 80/2478 and PPL 80/2050 which were reviewed and approved by the NIBSC animal ethical committee before submission. The experiments were performed under Home Office licence PPL 80/2060 and were specifically approved by the NIBSC animal ethics committee. The primates are gang housed with environmental enrichment including toys, swings, television. In the experiments described here the virus was administered in syrup that the animals had been trained to take willingly from a syringe (i.e. not by gavage); the animals were only housed alone when faecal samples were required. Anaesthesia and sacrifice at the end of the experiment were by scheduled methods as approved by the Home Office.

## Supporting Information

S1 Table Mutations found in S19 derived strains after ten passages of S19/N18S strains. (DOCX)
