# Supplementary material for: New Strains Intended for the Production of Inactivated Polio Vaccine at Low-Containment After Eradication
Source: PLoS Pathog. 2015 Dec 31;11(12):e1005316. doi: 10.1371/journal.ppat.1005316 (PMC4699825; doi:10.1371/journal.ppat.1005316)
Supplement: S1 Table — (DOCX) [file ppat.1005316.s001.docx]

| Virus strain | Cell line | 5’NCR | P1 | 2A |
| --- | --- | --- | --- | --- |
| S19/MahP1/N18S | MRC5  Vero | none  none | none  none | none  none |
| S19/MEF1P1/N18S | MRC5  Vero | none  128U-U/C | E1295K  E1295K | none  none |
| S19/SktP1/N18S | MRC5  Vero | none  none | none  E1094E/K N1144N/K | none  none |
| S19/S1P1/N18S | MRC5  Vero | none  none | Silent 1564U-U/C I11901I/V; D1298D/G  D1298-D/G/V/Y | none  none |
| S19/S2P1/N18S | MRC5  Vero | none  none | V3070A/V  I1143V/I;E1295K | none  none |
| S19/S3P1/N18S | MRC5  Vero | none  none | none  none | none  none |

S1 table. Mutations found after ten passages of S19/N18S strains
